# Hormonal regulation in adventitious roots and during their emergence under waterlogged conditions in wheat

**DOI:** 10.1093/jxb/ery190

**Published:** 2018-05-18

**Authors:** Tran-Nguyen Nguyen, Pham Anh Tuan, Shalini Mukherjee, SeungHyun Son, Belay T Ayele

**Affiliations:** Department of Plant Science, University of Manitoba, Winnipeg, Manitoba, Canada

**Keywords:** Adventitious roots, aerenchyma, gene expression, plant hormones, root elongation/growth, stem node, waterlogging

## Abstract

To gain insights into the molecular mechanisms underlying hormonal regulation in adventitious roots and during their emergence under waterlogged conditions in wheat, the present study investigated transcriptional regulation of genes related to hormone metabolism and transport in the root and stem node tissues. Waterlogging-induced inhibition of axile root elongation and lateral root formation, and promotion of surface adventitious and axile root emergence and aerenchyma formation are associated with enhanced expression levels of ethylene biosynthesis genes, *ACS7* and *ACO2*, in both tissues. Inhibition of axile root elongation is also related to increased root indole acetic acid (IAA) and jasmonate (JA) levels that are associated with up-regulation of specific IAA biosynthesis/transport (*TDC*, *YUC1*, and *PIN9*) and JA metabolism (*LOX8*, *AOS1*, *AOC1*, and *JAR1*) genes, and transcriptional alteration of gibberellin (GA) metabolism genes (*GA3ox2* and *GA2ox8*). Adventitious root emergence from waterlogged stem nodes is associated with increased levels of IAA and GA but decreased levels of cytokinin and abscisic acid (ABA), which are regulated through the expression of specific IAA biosynthesis/transport (*TDC*, *YUC1*, and *PIN9*), cytokinin metabolism (*IPT5-2*, *LOG1*, *CKX5*, and *ZOG2*), ABA biosynthesis (*NCED1* and *NCED2*), and GA metabolism (*GA3ox2* and *GA2ox8*) genes. These results enhance our understanding of the molecular mechanisms underlying the adaptive response of wheat to waterlogging.

## Teaser

Acclimation of wheat to waterlogging is mediated by regulation of hormonal metabolism and transport in adventitious roots and during their emergence.

## Introduction

Wheat (*Triticum aestivum* L.) is one of the most economically important cereal crops in the world; however, its production is challenged by a variety of abiotic stresses including waterlogging, which is reported to cause yield loss in ~15–20% of the wheat crop annually (Herzog *et al.*, 2016). Waterlogging causes significant reduction in gas diffusion and thereby creates low oxygen conditions in the root zone (Armstrong, 1979), and this reduces the synthesis of ATP and availability of carbohydrate resources for root growth and its functioning, leading to a substantial decrease in crop growth and yield (Herzog *et al.*, 2016).

Plants can acclimate to waterlogging-induced low oxygen conditions in the soil through morphological, anatomical, and metabolic responses. The major morphological/anatomical responses of roots of dryland plant species to oxygen-deficient conditions include the development of a shallow root system involving short and thick roots, and formation of aerenchymatous adventitious roots (Bailey-Serres *et al.*, 2012; Yamauchi *et al.*, 2018). Low oxygen conditions also cause inhibition of root growth/elongation and reduction in the emergence and elongation of lateral roots in wheat (Yamauchi *et al.*, 2014). Several studies have demonstrated that these root traits are controlled by plant hormones. For example, ethylene, auxin, and abscisic acid (ABA) are involved in the inhibition of primary root elongation in plant species such as rice and wheat under normal/non-stress conditions (Konishi *et al.*, 2005; McSteen, 2010; Yu *et al.*, 2016), while gibberellin (GA) is implicated in promoting root elongation (Komatsu and Konishi, 2005). Previous reports, however, have shown the requirement of ABA for maintenance of primary root growth under stress conditions such as drought/low water potential (Saab *et al.*, 1990; Sharp *et al.*, 1994). Other plant hormones such as cytokinin and jasmonates (JAs) are also reported to inhibit primary root growth (Stenlid, 1982; Liu *et al.*, 2015). Previous studies have also implicated ethylene in inducing aerenchyma formation in the root of dryland plant species such as wheat and maize under oxygen-deficient conditions through enhancing cortical cell death (Jackson *et al.*, 1985; Jackson and Armstrong, 1999; He *et al.*, 1994, 1996; Yamauchi *et al.*, 2014).

Adventitious roots that emerge from stem nodes in response to waterlogging often develop aerenchyma connected to the shoot, improving gas diffusivity along the root (Sauter, 2013; Steffens and Rasmussen, 2016). Several studies have shown that auxin plays a key role in adventitious root formation (Delarue *et al.*, 1998; Falasca and Altamura, 2003; Vidoz *et al.*, 2010). Adventitious roots emerge through the nodal epidermis, and this process is enhanced by ethylene through its action of inducing nodal epidermal cell death (Mergemann and Sauter, 2000). Ethylene-induced death of epidermal cells has been shown to be mediated by reactive oxygen species (ROS) (Steffens *et al.*, 2012; Gonzali *et al.*, 2015; Mittler, 2017). Furthermore, ethylene-induced death of cortical cells, which leads to aerenchyma formation, is reported to be mediated by ROS (Yamauchi *et al.*, 2017). Other plant hormones such as cytokinin, JA, ABA, and GA are also implicated in regulating adventitious root formation (Verstraeten *et al.*, 2014), highlighting the significance of finely regulated hormonal crosstalk. For example, auxin regulates adventitious root formation by modulating JA homeostasis through auxin-inducible Gretchen Hagen 3 (GH3)-like proteins (Gutierrez *et al.*, 2012), while the role of ethylene in regulating adventitious root formation can also be associated with its regulation of auxin transport (Vidoz *et al.*, 2010). Cytokinin is reported to act as an auxin antagonist in the formation of adventitious roots, probably through inducing Aux/IAA (auxin/indole acetic acid), and thereby repressing PIN proteins and auxin flow (Della Rovere *et al.*, 2013). It has also been shown that GA promotes ethylene-induced adventitious root formation while its antagonist ABA represses ethylene-induced and GA-promoted emergence of adventitious roots (Steffens *et al.*, 2006).

Despite these reports, the molecular mechanisms underlying the regulation of hormonal metabolic pathways in adventitious roots and during their emergence in response to waterlogging are poorly understood in dry land crop species, especially in the polyploid wheat for which genomic/genetic resources to undertake such studies have been limited. To this end, the present study integrated physiological, histological, molecular, and metabolomics approaches to investigate changes in root anatomy/morphology, expression patterns of hormonal metabolic genes, and the levels of the respective plant hormones in wheat root and stem node tissues exposed to waterlogging during the stem elongation growth phase, which is reported to be very sensitive to waterlogging-induced yield losses (Marti *et al.*, 2015).

## Materials and methods

### Plant growth and waterlogging treatment

Plants of common wheat cv. Harvest were grown in 3 liter plastic pots (one plant per pot) containing a mixture of locally sourced top soil and sand (2:1, v:v) supplied with fertilizers at 22 °C/20 °C (day/night) under a 16 h/8 h photoperiod as described previously (Nguyen *et al.*, 2016). The top soil comprised 39% sand, 17% silt, and 44% clay, while the sand soil comprised 98% sand, 1% silt, and 1% clay. Thirty-day-old plants were subjected to waterlogging by submerging each pot in a 6 liter pot filled with water in order to maintain the water level at ~2 cm above the soil surface, and more water was added to the bigger pot as needed to maintain the water level. Control plants were subjected to regular watering (0.5 liter per pot every other day). At 1, 7, 14, and 28 days after waterlogging (DAWL), each pot containing the waterlogged or control plant was completely immersed in a large (63 liter) plastic box filled with tap water to loosen and remove the soil from the pot. Roots were separated gently from the soil while still in the water (for a maximum of 10 min), followed by a final wash of roots with a gentle stream of tap water to clean any remaining soil particles (for a maximum of 5 min). Control and waterlogged basal stem nodes, located within the 2 cm of water above the soil surface and from which new adventitious roots (hereafter referred to as ‘surface adventitious roots’) emerged, were excised from the main stem and tillers. Stem nodes were collected from plants waterlogged for 7, 14, and 28 d and their respective controls as they were not detected in most of the tillers/stems at 1 DAWL. To determine gene expression and hormone levels, three independent biological replicates of the whole root (roots of an individual plant/replicate), which consists of a mixture of the various root types described above (hereafter referred to as ‘root’), and stem node (stem nodes of individual plant/replicate) tissues were collected from both control and waterlogged plants. Tissues were immediately fixed (root tissues), or frozen in liquid nitrogen (roots and stem nodes) and then stored at –80 °C until further use. Root and shoot dry weights, number of tillers, number and length of axile (primary and nodal) roots, number of branch/lateral roots (see Chochois *et al.*, 2012 for a description of root types), and number of surface adventitious roots were determined manually using freshly harvested roots (roots of three individual plants per replicate, three replicates).

### Identification of wheat hormone metabolic genes

The hormone metabolism genes analyzed in this study were selected from our preliminary GenChip analysis based on the differential expression (≥2-fold) of their respective probe sets between control and waterlogged roots at 14 and/or 28 DAWL. To annotate the probe sets, sequences of the hormone metabolic genes of Arabidopsis were first identified from The Arabidopsis Information Resource database and used as queries to search for their homologs in the Rice Genome Annotation Project database (http://rice.plantbiology.msu.edu/) using an E- value of <10^−10^. The resulting rice gene sequences were then blast searched against the wheat 61 k microarray platform in the Plant Expression Database (PLEXdb; http://www.plexdb.org/) to identify the corresponding probe sets and wheat unigenes using an E-value of 10^–20^. Sequences of the wheat unigenes were then blast searched against the wheat genome data in Ensembl Plants (http://plants.ensembl.org/Triticum_aestivum/) to identify the respective full-length wheat genes and proteins, which were used as queries to search for orthologs in Arabidopsis and other cereal species (Supplementary Table S1 at *JXB* online). Genes/homologs were named based on their orthologs in Arabidopsis and/or other cereal species (Supplementary Table S1), and the naming of the cytokinin metabolism and IAA transport genes has taken into account previously published reports (Song *et al.*, 2012; Talboys *et al.*, 2014). Genes related to ROS production, and ABA and GA (*GA3ox2* and *GA3ox3*) metabolism have been reported previously (see Supplementary Table S2 for the references). Primer information of the genes analyzed in this study is presented in Supplementary Table S2.

### RNA extraction and quantitative (q) RT-PCR analysis

Total RNA was extracted from the root and the stem node tissues using Trizol (Invitrogen, Carlsbad, CA, USA), and the cDNA samples for qPCR assays were prepared as described previously (Nguyen *et al.*, 2016). Relative transcript levels were determined using the Livak and Schmittgen (2001) method after normalization with *18SrRNA* as a reference gene. Transcript levels were compared across gene homologs, tissues, and growth conditions using the average transcript levels of one of the homologs in control roots at 1 DAWL as a reference sample (see figure legends for the details). For accurate and reproducible comparisons of the expression levels among the gene homologs, qRT-PCR efficiency of each gene was determined using the Ct slope method as described previously (Yao *et al.*, 2012), and amplification efficiencies within the 90–105% acceptable range were obtained for all the genes studied (Supplementary Table S2).

### Anatomical analysis of aerenchyma in the root

Nodal root segments (at 4 cm from the root apex) from plants waterlogged for 28 d and the respective controls were collected directly in a fixative containing 2.5% glutaraldehyde and 1.6% paraformaldehyde in 0.05 M phosphate-buffered saline (PBS). The samples were subjected to a vacuum for 30 min at 25 mm of Hg and then stored overnight at 4 °C. The fixed tissues were then serially dehydrated in 50, 70, 95, and 100% ethanol for 90 min in each solution at 4 °C. The dehydrated tissues were transferred to a mixture of absolute ethanol:historesin (2:1, v/v) containing the activator (5 g in 50 ml) and incubated for 24 h followed by a transfer to a mixture of absolute ethanol:historesin (1:2, v/v) and incubation for 24 h, and finally to 100% historesin for 48 h in which a freshly prepared solution was used after the first 24 h. After embedding in glycol methacrylate, sections (4 μm) were prepared using a JB-4 Sorvall microtome (Thermo Scientific, Waltham, MA, USA) and then stained for 2 min in 0.05% (w/v) toluidine blue O in benzoate buffer at pH 4.4. After destaining and drying, the samples were examined with an Olympus CX41 bright field microscope (Olympus Life Science, Tokyo, Japan) at ×4. The images of the sections were captured using a Lumenera Infinity HD camera (Lumenera Corporation, Ottawa, ON, Canada).

### Hormone measurement

Lyophilized root and stem node tissues harvested from control and waterlogged plants were homogenized with acetonitrile containing 1% (v/v) acetic acid and internal standards as described previously (Son *et al.*, 2016; Izydorczyk *et al.*, 2018). Extraction of acidic and basic hormones from the homogenates and subsequent analyses of their levels with LC-ESI-MS/MS (Agilent 1260-6430) were performed as described in Yoshimoto *et al.* (2009).

### Statistical analysis

Statistically significant differences between control and waterlogged samples were tested by Student’s *t*-test at a probability of *P*<0.05.

## Results

### Root and shoot growth, anatomy, and morphology

**Fig. 1.**
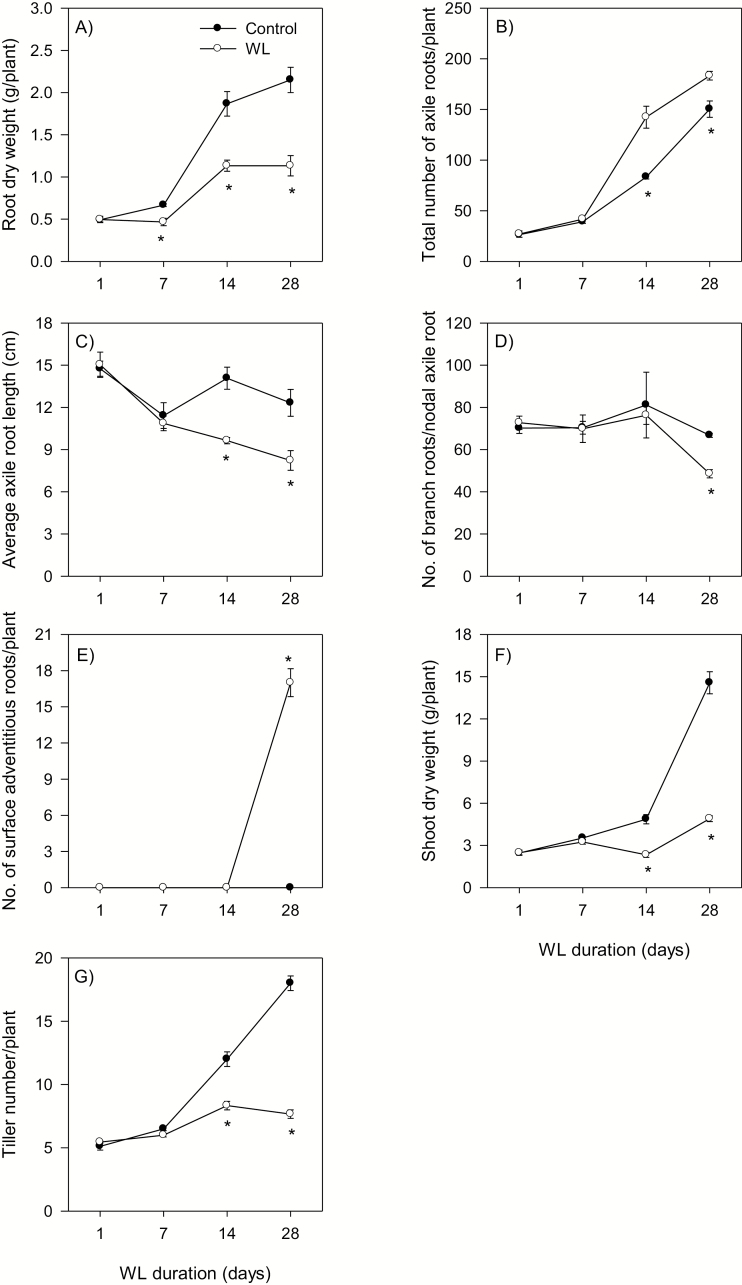
Root and shoot phenotype under control and waterlogged conditions. Root dry weight (A), total number of axile roots per plant (B), average length of axile root (C), number of branch roots per nodal axile root (D), number of surface adventitious roots per plant (E), shoot dry weight (F), and tiller number (G) of plants waterlogged for 1, 7, 14, and 28 d, and their respective controls. Data are means of three independent replicates (roots of three individual plants per replicate) ±SE. Asterisks denote a statistically significant difference between samples derived from control and waterlogged plants within each duration of waterlogging (*P*<0.05; Student *t*-test). WL, waterlogged.

**Fig. 2.**
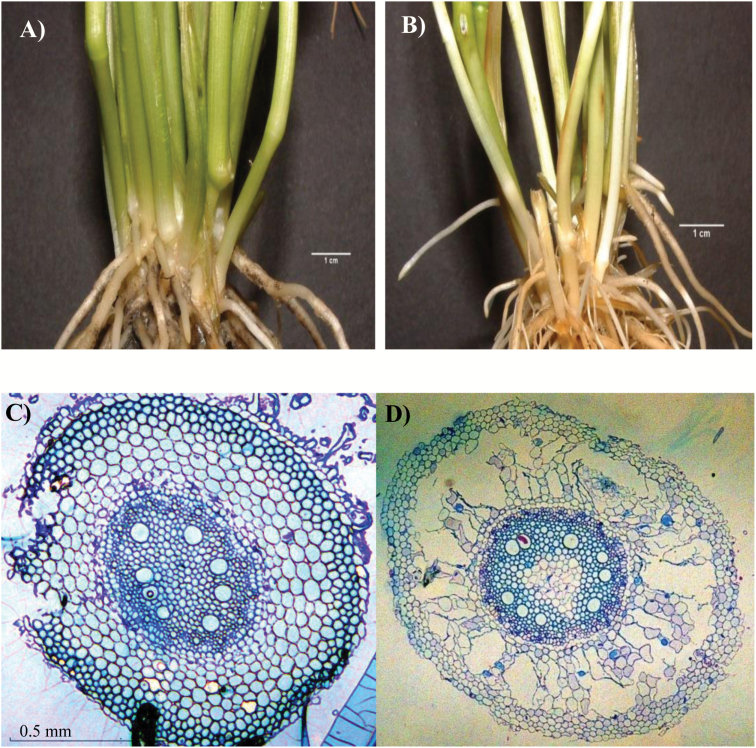
Emergence of surface adventitious root and aerenchyma formation. Comparison of surface adventitious root emergence in control (A) and waterlogged (B) plants at 28 d after waterlogging. Lateral sections of control (C) and 28 d waterlogged (D) nodal root segments (4 cm from the root apex) at ×4 magnification. Aerenchyma formation is evident in the cortical cells of roots waterlogged for 28 d.

Waterlogging for 7 d caused a 30% reduction in root dry weight, and this effect was more pronounced as waterlogging continued for 14 d and 28 d (40–48%) (Fig. 1A). Waterlogging for 14 d and 28 d increased the total number of axile roots (primary and nodal) per plant but decreased their average length (Fig. 1B, C). Reduction in the number of branch roots per nodal axile root, as determined from the five longest nodal axile roots, was observed only after 28 d of waterlogging (Fig. 1D). Formation of surface adventitious roots (17 per plant) was also evident only after 28 d of waterlogging (Figs 1E, 2B), and this was accompanied by aerenchyma formation in the nodal axile roots (Fig. 2D). Waterlogging for 14 d and 28 d also decreased tiller number (1.5- to 2.3-fold) and shoot dry weight (52–66%) (Fig. 1F, G).

### Transcriptional regulation of genes related to ethylene biosynthesis

Ethylene is synthesized from *S*-adenosylmethionine, which is converted to 1-aminocyclopropane-1-carboxylic acid (ACC) by the action of ACC synthase (ACS) (Yang and Hoffman, 1984). This reaction represents the first committed and rate-limiting step of ethylene synthesis. Subsequently a reaction catalyzed by ACC oxidase (ACO) converts ACC to ethylene. Our study examined the expression patterns of *ACS* genes (*ACS2*, *ACS4*, and *ACS7*) and *ACO* genes (*ACO2*, *ACO4*, and *ACO5*) in both root and stem node tissues under control and waterlogged conditions. The expression levels of *ACS4*, *ACS7*, *ACO2*, and *ACO4* were relatively higher than those of the other homologs in the root and/or stem node tissues under both growth conditions (Supplementary Table S3).

#### Root

**Fig. 3.**
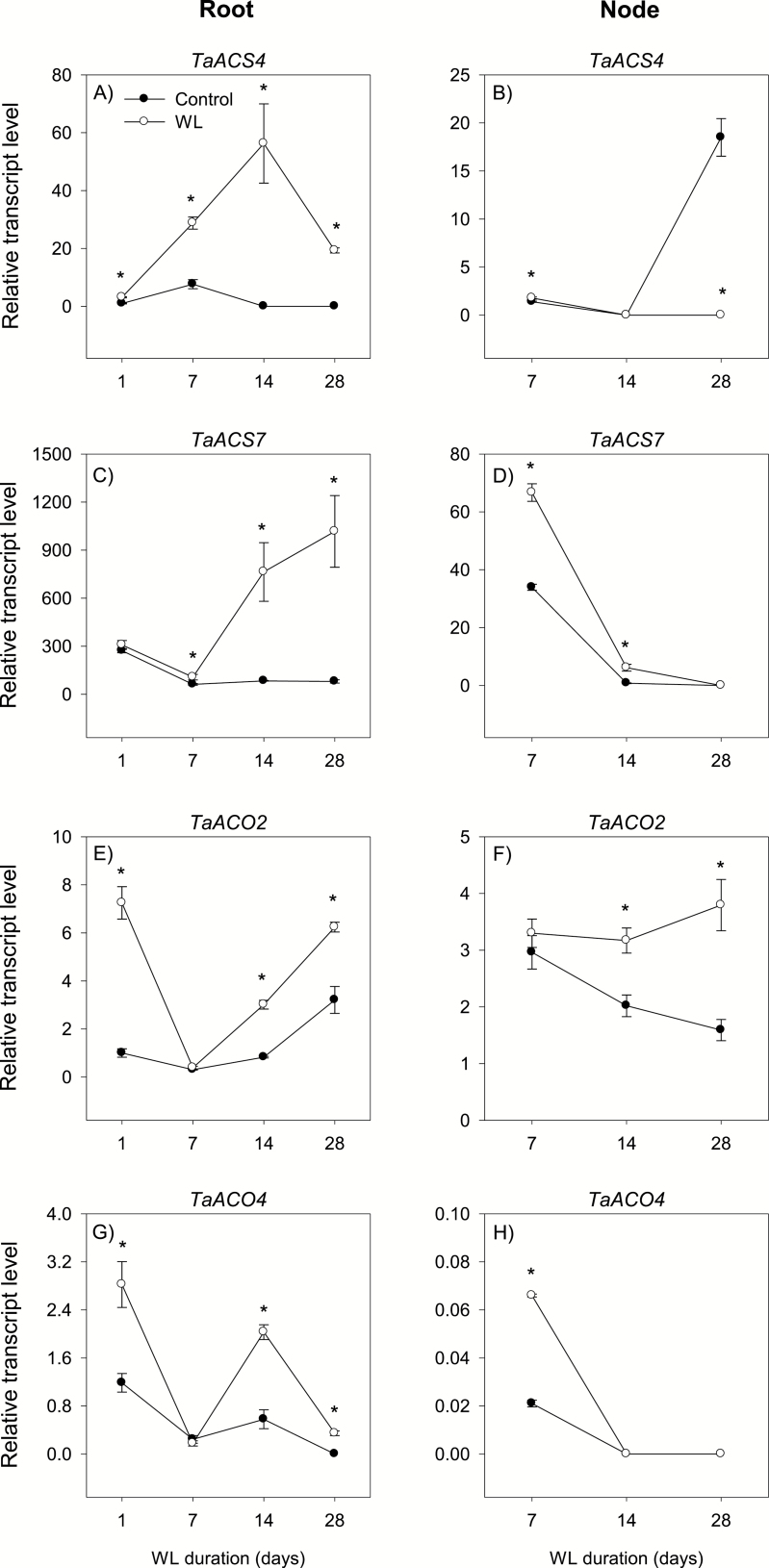
Expression of ethylene biosynthesis genes. Relative transcript levels of *ACS4* (A and B), *ACS7* (C and D), *ACO2* (E and F), and *ACO4* (G and H) in the root tissue waterlogged for 1, 7, 14, and 28 d (A, C, E, and G) and stem node tissue waterlogged for 7, 14, and 28 d (B, D, F, H), and their respective controls. Transcript levels of the *ACS* and *ACO* genes in both tissues were determined using *Ta18SrRNA* as the reference gene and expressed relative to the transcript levels of *ACS4* and *ACO2* in control roots at 1 d after the start of waterlogging, respectively, which were arbitrarily set a value of 1. Data are means of three biological replicates ±SE. Asterisks denote a statistically significant difference in transcript levels between tissue samples derived from control and waterlogged plants within each duration of waterlogging (*P*<0.05; Student *t*-test). WL, waterlogging. *ACS2* and *ACO5* exhibited either very minimal or undetectable levels of expression irrespective of the tissue type or growth conditions, as shown in Supplementary Table S3.

The expression levels of *ACS4* and *ACS7* in the root decreased to low/below detectable levels during the course of the experiment under control conditions but increased in response to waterlogging for 1 d (*ACS4*; >3-fold) or 7 d (*ACS7*; >1.7-fold) (Fig. 3A, C). The expression levels of *ACO2* and *ACO4* were constantly very weak under control conditions, except for the transient increase of *ACO2* expression at 28 DAWL (Fig. 3E, G). Waterlogging for 1, 14, and 28 d increased the expression levels of both genes (~2-fold or more). Although the expression of *ACO5* was very weak in the root under control conditions, waterlogging for 14 d and 28 d enhanced its expression level (Supplementary Table S3). Under both growth conditions, the expression levels of *ACS7* and *ACO2* were higher than those of the other homologs.

#### Stem node

The expression of *ACS4* and *ACS7* in the stem node decreased below detectable levels by the end of the experiment under both control and waterlogged conditions, except for the marked increase of *ACS4* expression in control stem nodes at 28 DAWL (Fig. 3B, D). Both genes, however, exhibited higher levels of expression in stem nodes waterlogged for 7 d and/or 14 d (*ACS4*, 1.3-fold; *ACS7*, ~2-fold or more) than the respective controls. Stem nodes waterlogged for 7 d and the corresponding controls exhibited a similar level of *ACO2* expression, and this level was maintained with further waterlogging but decreased under control conditions (1.9-fold) (Fig. 3F). The expression level of *ACO4* in the stem node was either very minimal or below the detectable level under both growth conditions (Fig. 3H). Overall, *ACS7* and *ACO2* exhibited the highest levels of expression in the stem node irrespective of growth conditions.

### Transcriptional control of genes related to ROS production

ROS are produced as by-products of basic cellular processes such as photosynthesis and respiration, and through the action of several enzymes. Respiratory burst oxidase homologs (RBOHs), also known as NADPH oxidases, have been reported to be key players in this regard (Suzuki *et al.*, 2011). Our study examined the expression patterns of three *RBOH* genes (*RBOH1*, *RBOH2*, and *RBOH3*) in the root and stem node tissues under control and waterlogged conditions.

#### Root

**Fig. 4.**
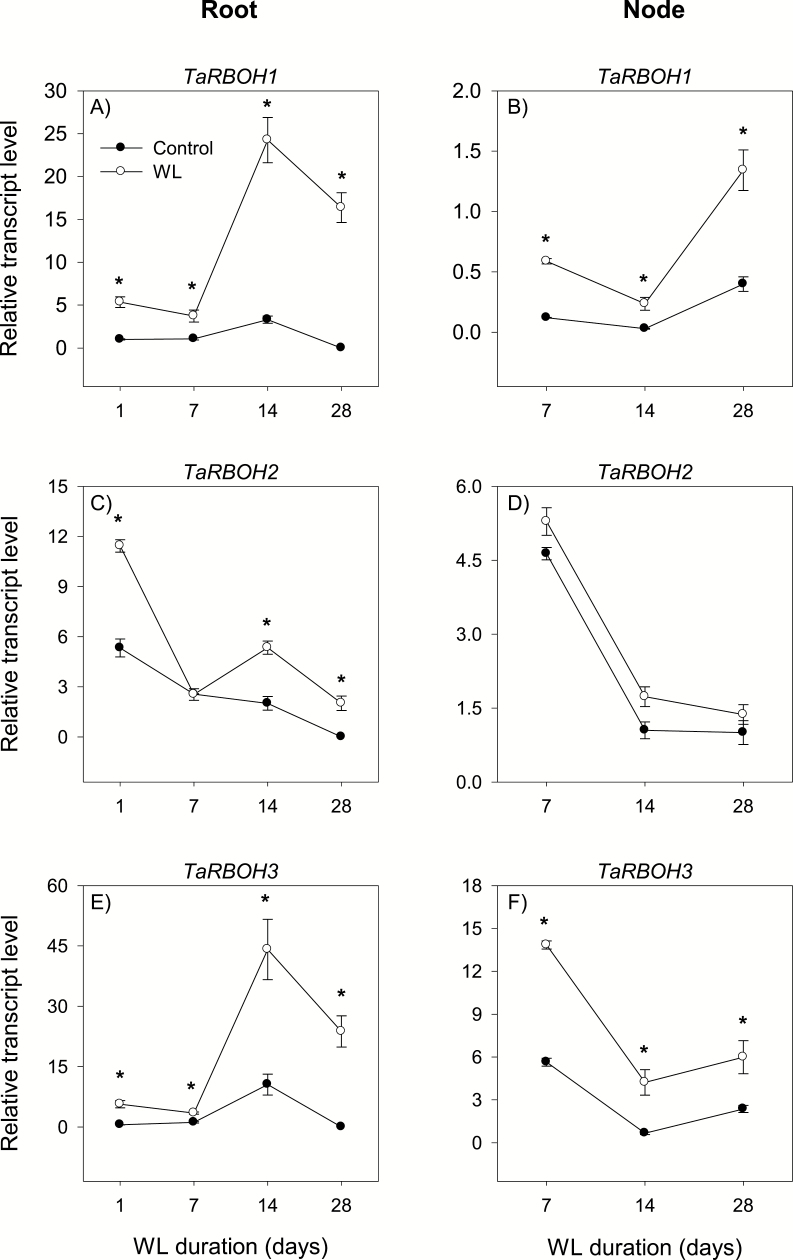
Expression of respiratory burst oxidase homolog genes. Relative transcript levels of *RBOH1* (A and B), *RBOH2* (C and D), and *RBOH3* (E and F) in the root tissue waterlogged for 1, 7, 14, and 28 d (A, C, and E) and stem node tissue waterlogged for 7, 14, and 28 d (B, D, and F), and their respective controls. Transcript levels of each gene in both tissues were determined as described in Fig. 3, and are expressed relative to the transcript level of *RBOH1* in control roots at 1 d after the start of waterlogging, which was arbitrarily set a value of 1. Other data descriptions are as indicated in Fig. 3.

The expression of all *RBOH* genes in the root decreased below detectable levels by the end of the experiment under control conditions despite the transient increases observed for *RBOH1* and *RBOH3* at 14 DAWL (Fig. 4A, C, E). Waterlogging, however, increased the expression levels of *RBOH* genes (from undetectable levels or >2-fold), with the major effects observed at 14 and 28 DAWL.

#### Stem node

The expression levels of all *RBOH* genes in the stem node either decreased to low levels or remained constantly low during the course of the experiment under control conditions (Fig. 4B, D, F). Waterlogging increased the expression levels of *RBOH1* and *RBOH3* (>2.5-fold) but did not affect the expression level of *RBOH2*.

### Regulation of IAA metabolism and transport

IAA is the major bioactive auxin in plants, and it is produced from tryptophan via indole-3-pyruvate by the actions of tryptophan aminotransferase (TAA) and YUCCAs (YUCs), respectively (Kasahara, 2016). Alternatively, tryptophan is proposed to be converted to IAA via tryptamine and indole-3-acetaldehyde by tryptophan decarboxylase (TDC) and aldehyde oxidase (AAO), respectively (Quittenden *et al.*, 2009). IAA is inactivated mainly through its conjugation with amino acids by the action of GH3 proteins. The IAA level in plants is also regulated by its transport, which is mediated by the PIN FORMED (PIN) auxin efflux carriers that have a polar cellular distribution. This study examined the expression patterns of IAA biosynthesis (*TDC*, *AAO*, *TAA1*, *YUC1*, and *YUC10*), conjugation (*GH3*; *GH3.1*, and *GH3.2*), and transport (*PIN*; *PIN5*, and *PIN9*) genes in waterlogged root and stem node tissues and their respective controls. Our analysis revealed that the *TDC*, *YUC1*, *GH3.1*, *GH3.2*, and *PIN9* are expressed in the root and/or stem node tissues under both control and waterlogged conditions, while no transcript of *TAA1*, *AAO*, *YUC10*, and *PIN5* was detected irrespective of tissue type or growth conditions (Supplementary Table S3).

#### Root

**Fig. 5.**
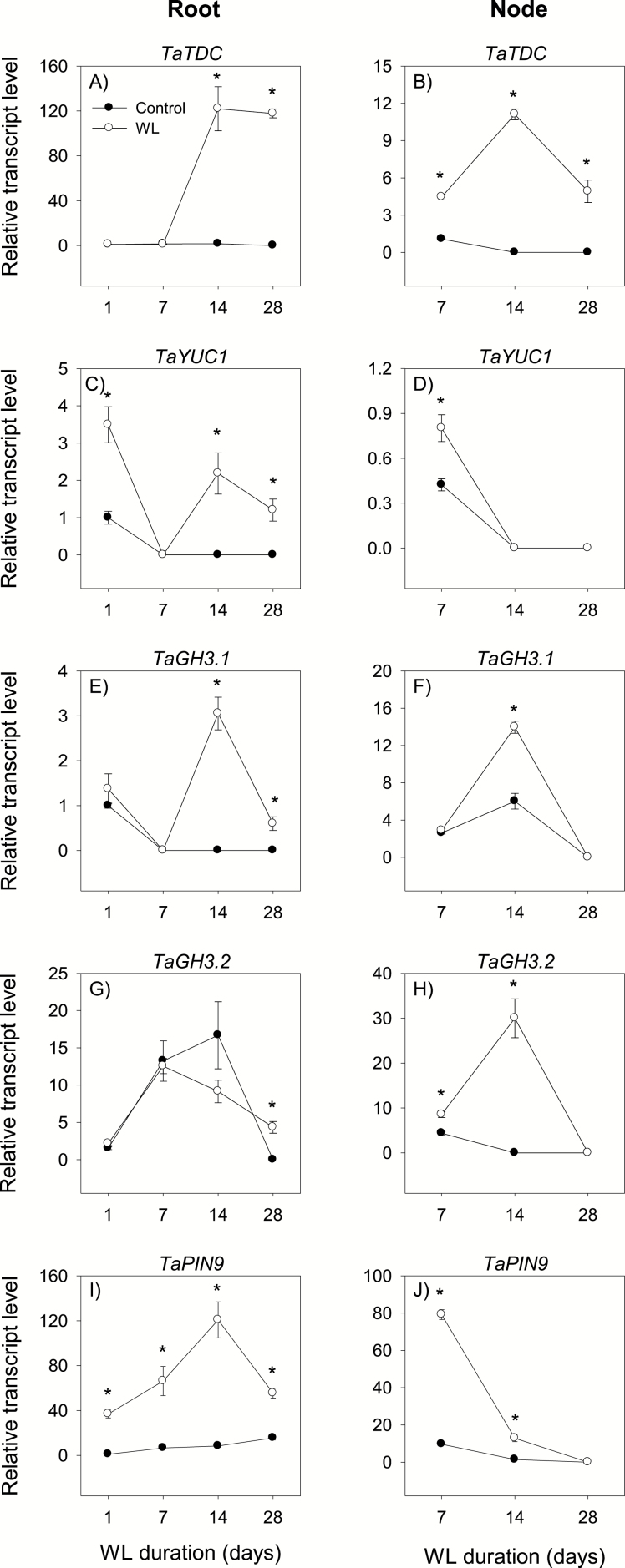
Expression of auxin metabolism and transport genes. Relative transcript levels of *TDC* (A and B), *YUC1* (C and D), *GH3.1* (E and F), *GH3.2* (G and H), and *PIN9* (I and J) in root tissue waterlogged for 1, 7, 14, and 28 d (A, C, E, G, and I) and stem node tissue waterlogged for 7, 14, and 28 d (B, D, F, H, and J), and their respective controls. Transcript levels of *TDC*, *YUC1*, *GH3*, and *PIN9* genes in both tissues were determined as described in Fig. 3, and are expressed relative to the respective transcript levels of *TDC*, *YUC1*, *GH3.1*, and *PIN9* in control roots at 1 d after the start of waterlogging, respectively, which were arbitrarily set a value of 1. Other data descriptions are as indicated in Fig. 3. No transcript of *TAA1*, *AAO*, *YUC10*, and *PIN5* was detected irrespective of tissue type or growth conditions, as shown in Supplementary Table S3.

**Fig. 6.**
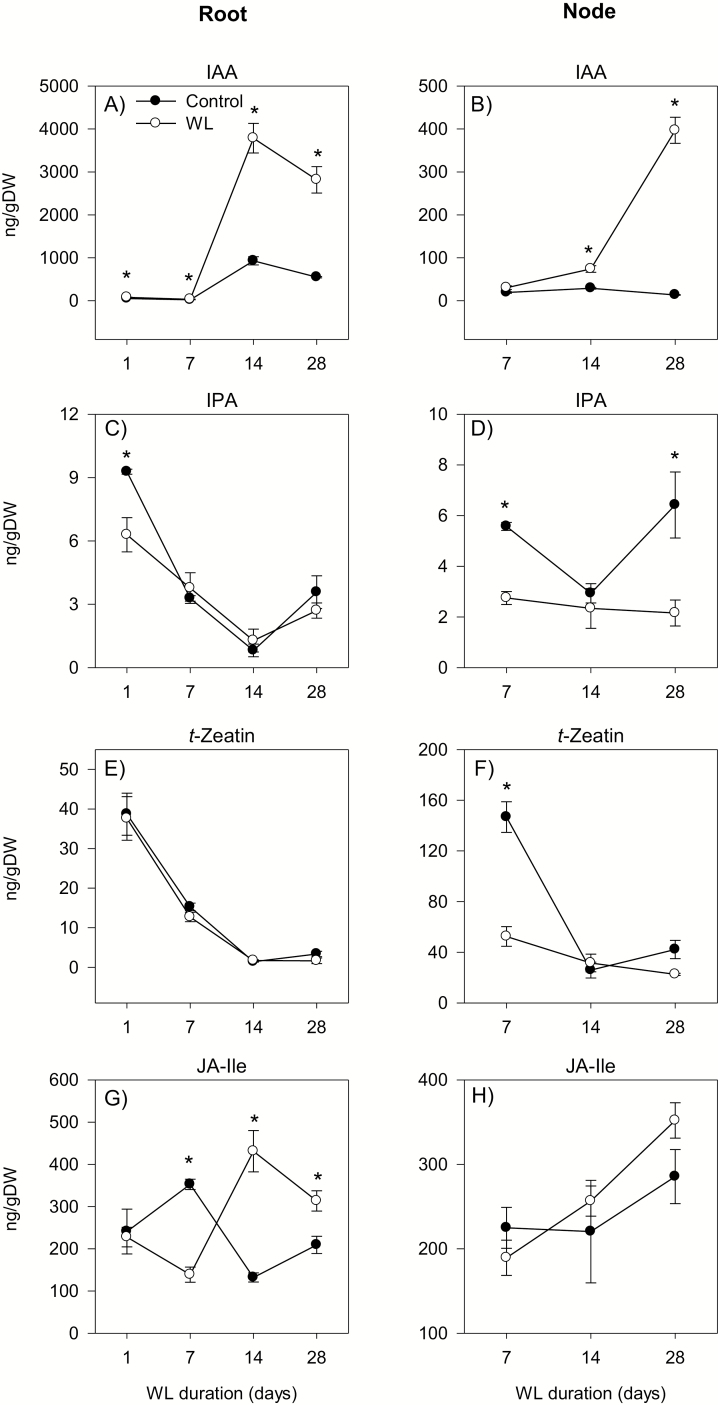
Auxin, cytokinin, and jasmonate levels. Indole acetic acid (IAA; A and B), *N*^6^-isopentenyladenine (IPA; C and D), *trans*-zeatin (*t*-zeatin, E and F), and jasmonoyl-l-isoleucine (JA-Ile; G and H) levels in the root tissue waterlogged for 1, 7, 14, and 28 d (A, C, E, and G) and stem node tissue waterlogged for 7, 14, and 28 d (B, D, F, and H), and their respective controls. Data are means of three biological replicates ±SE. Asterisks denote a statistically significant difference in hormone levels between samples derived from control and waterlogged plants within each duration of waterlogging (*P*<0.05; Student *t*-test). WL, waterlogging.

The expression of *TDC* and *YUC1* in the root decreased below detectable levels during the course/by the end of the experiment under control conditions but increased by waterlogging for 14 d and 28 d (Fig. 5A, C). The expression level of *YUC1* also increased by waterlogging for 1 d (3.5-fold). The expression level of *PIN9* was constantly very weak under control conditions but increased (3.5- to 37-fold) by waterlogging irrespective of the duration (Fig. 5I). Waterlogging for 14 d and/or 28 d also increased the expression levels of *GH3.1* and *GH3.2* (from undetectable levels) (Fig. 5E, J). The root IAA level increased under both control and waterlogged conditions; however, roots waterlogged for 14 d and 28 d exhibited a higher (>4-fold) level of IAA than that observed in the respective controls (Fig. 6A).

#### Stem node

The expression of *TDC* in the stem node decreased below a detectable level during the course of the experiment under control conditions but increased (from undetectable levels or >4-fold) by waterlogging irrespective of the duration (Fig. 5B). Stem nodes waterlogged for 7 d and/or 14 d exhibited higher expression levels of *YUC1* and *PIN9* (~2-fold or more) than that observed in the corresponding controls (Fig. 5D, J). The expression of both genes decreased below detectable levels by 28 DAWL under both growth conditions. The expression of *GH3.1* and *GH3.2* also decreased below detectable levels under both growth conditions, except for the strong transient increases of their expression by waterlogging for 14 d (Fig. 5F, H). An increase in the expression level of *GH3.2* was also observed by waterlogging for 1 d. The stem node IAA level remained constant under control conditions, but increased in response to waterlogging for 14 d and 28 d (2.5- to 29-fold) (Fig. 6B).

### Regulation of cytokinin metabolism

The first and rate-limiting step of cytokinin biosynthesis involves the formation of isopentenyladenine (iP) nucleotides from dimethylallyl diphosphate and adenine nucleotides, preferably ATP and ADP, by the action of adenosine phosphate-isopentenyltransferase (IPT) (Hirose *et al.*, 2008). Cytochrome P450 monooxygenases (CYP735As) convert the iP nucleotides to *trans*-zeatin (*t*-zeatin) nucleotides; both the iP and *t*-zeatin nucleotides can be directly activated to free base bioactive cytokinins by cytokinin nucleoside 5′-monophosphate phosphoribohydrolase, also known as lonely guy (LOG) (Kurakawa *et al.*, 2007). Inactivation of cytokinin is catalyzed by cytokinin oxidases/dehydrogenases (CKXs) and/or cytokinin-conjugating *O*-glucosyltransferases (ZOGs). In this study, we analyzed the expression patterns of cytokinin biosynthesis (*IPT2-2* and *IPT5-2*), activation (*LOG1*, *LOG3*, and *LOG5*), catabolism (*CKX4-2* and *CKX5*), and conjugation (*ZOG2* and *cZOG2-3*) genes in waterlogged root and stem node tissues and their respective controls. Our data revealed that all the cytokinin metabolic genes studied are expressed in the root and stem node tissues under both control and waterlogged conditions, except that the expression levels of *LOG3* in both tissues, and *IPT2-2* and *LOG5* in the stem node tissue were very minimal/undetectable irrespective of growth conditions (Supplementary Table S3).

#### Root

**Fig. 7.**
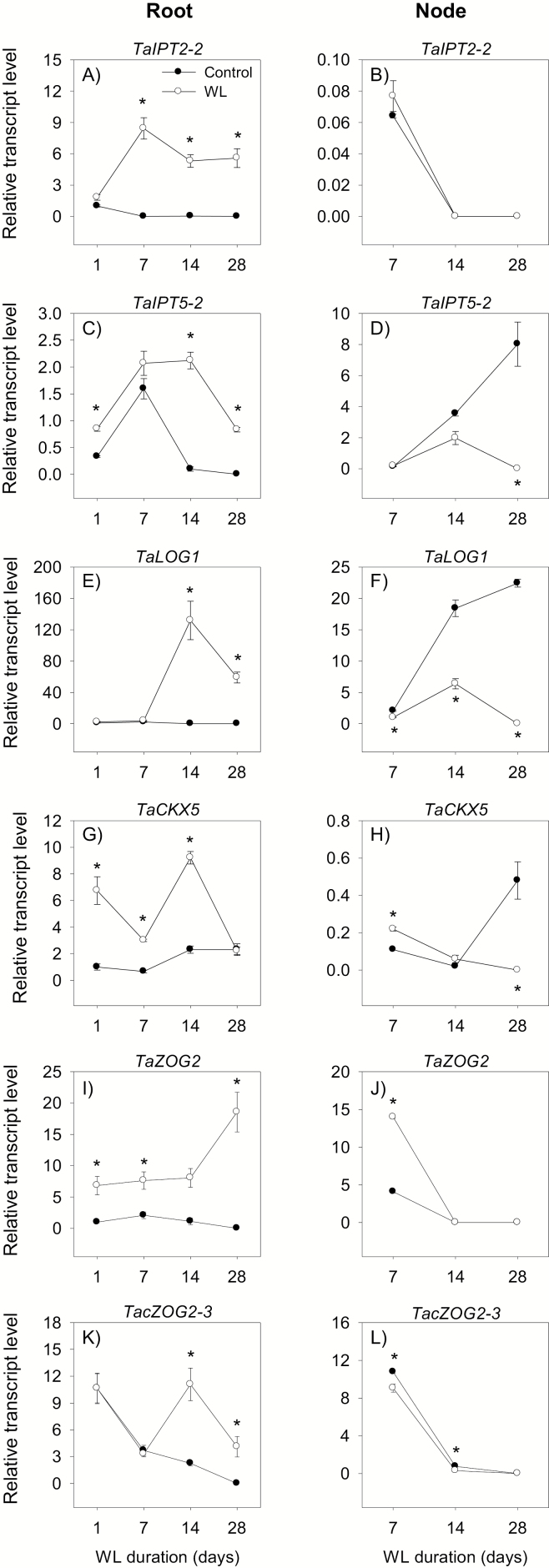
Expression of cytokinin metabolism genes. Relative transcript levels of *IPT2-2* (A and B), *IPT5-2* (C and D), *LOG1* (E and F), *CKX5* (G and H), *ZOG2* (I and J), and *cZOG2-3* (K and L) in the root tissue waterlogged for 1, 7, 14, and 28 d (A, C, E, G, I, and K) and stem node tissue waterlogged for 7, 14, and 28 d (B, D, F, H, J, and L), and their respective controls. Transcript levels of *IPT* genes, *LOG1*, *CKX5*, and *ZOG* genes in both tissues were determined as described in Fig. 3, and are expressed relative to the transcript levels of *IPT2-2*, *LOG1*, *CKX5*, and *ZOG2* in control roots at 1 d after the start of waterlogging, respectively, which were arbitrarily set a value of 1. Other data descriptions are as indicated in Fig. 3. Expression levels of *LOG3* in both tissues, and *IPT2-2* and *LOG5* in the stem node were either minimal or undetectable irrespective of growth conditions, as shown in Supplementary Table S3.

The expression levels of *IPT2-2*, *IPT5-2*, *LOG1*, and *LOG5* in the root decreased below detectable levels during the course or by the end of the experiment under control conditions but strongly increased in response to waterlogging for seven and/or more days (Fig. 7A, C, E; Supplementary Table S3). Between the two *IPT* genes, *IPT2-2* exhibited the highest level of waterlogging-induced expression. Under control conditions, the expression level of the cytokinin inactivation gene, *CKX5*, increased (>2-fold) while the expression levels of *CKX4-2*, *ZOG2*, and c*ZOG2-3* decreased below detectable levels (Fig. 7G, I, K; Supplementary Table S3). Waterlogging, however, led to temporally distinct increases in the expression levels of these genes; *CKX5* expression level was induced by waterlogging up to 14 d while the expression levels of the other genes were induced by waterlogging irrespective of the duration (*ZOG2*) or mainly at 14 and 28 DAWL (*CKX4-1* and *ZOG2-3*). Root *N*^6^-isopentenyladenine (IPA) and *t*-zeatin levels decreased under both control and waterlogged conditions, and were not affected by waterlogging (Fig. 6C, E).

#### Stem node

The expression level of *IPT2-2* in the stem node was very minimal or decreased below a detectable level during the course of the experiment under both control and waterlogged conditions (Fig. 7B). The expression levels of *IPT5-2* and *LOG1*, however, increased under control conditions but decreased (>1.8-fold or to undetectable levels) by waterlogging for 14 d and 28 d (*IPT5-2*) or irrespective of the duration (*LOG1*) (Fig. 7D, F). Between the two *IPT* genes, *IPT5-2* showed the highest level of expression under both growth conditions. Expression levels of cytokinin-inactivating genes (*CKX4-2*, *CKX5*, *ZOG2*, and *cZOG2-3*) decreased to lower or below detectable levels under both growth conditions, except for the increase of *CKX5* expression in control stem nodes at 28 DAWL (Fig. 7H, J, L; Supplementary Table S3). However, stem nodes waterlogged for 7 d exhibited higher expression levels of *CKX4-2*, *CKX5*, and *ZOG2* (>2-fold) than that observed in their respective controls. The levels of IPA and *t*-zeatin in the stem node decreased/slightly decreased under both growth conditions, except for the increase of IPA level in control stem nodes at 28 DAWL (to a level similar to that observed at 7 DAWL) (Fig. 6D, F). Stem nodes waterlogged for 7 d and 28 d, however, contained a lower amount of IPA and *t*-zeatin (>2-fold) than that found in their respective controls.

### Regulation of jasmonate metabolism

The synthesis of JA involves the conversion of polyunsaturated fatty acids to 12-oxo-phytodienoic acid (OPDA) by a series of reactions catalyzed by lipoxygenase (LOX), allen oxide synthase (AOS), and allen oxide cyclase (AOC) (Schaller and Stintzi, 2009). The OPDA is then converted to jasmonic acid by the action of OPDA reductase (OPR) and three cycles of β-oxidation. Jasmonate resistant 1 (JAR1), which is a jasmonate-amido synthetase, catalyzes the conversion of jasmonic acid to jasmonyl-l-isoleucine conjugate (JA-Ile), a type of JA that is crucial for JA signaling (Staswick, 2008). This study analyzed the expression patterns of genes involved in JA biosynthesis including *LOX* (*LOX2* and *LOX8*), *AOS* (*AOS1* and *AOS2*), and *AOC* (*AOC1* and *AOC2*), and JA conjugation (*JAR*; *JAR1*) in waterlogged root and stem node tissues and their respective controls. Our analysis revealed that all the JA metabolic genes studied are expressed in both root and stem node tissues under control and/or waterlogged conditions except that the expression of *LOX2* and *AOS2* in the root and *AOC2* in the stem node was either below detectable levels or relatively lower than the levels of their other homologs (*LOX8*, *AOS1*, and *AOC1*) irrespective of growth conditions (Supplementary Table S3).

#### Root

**Fig. 8.**
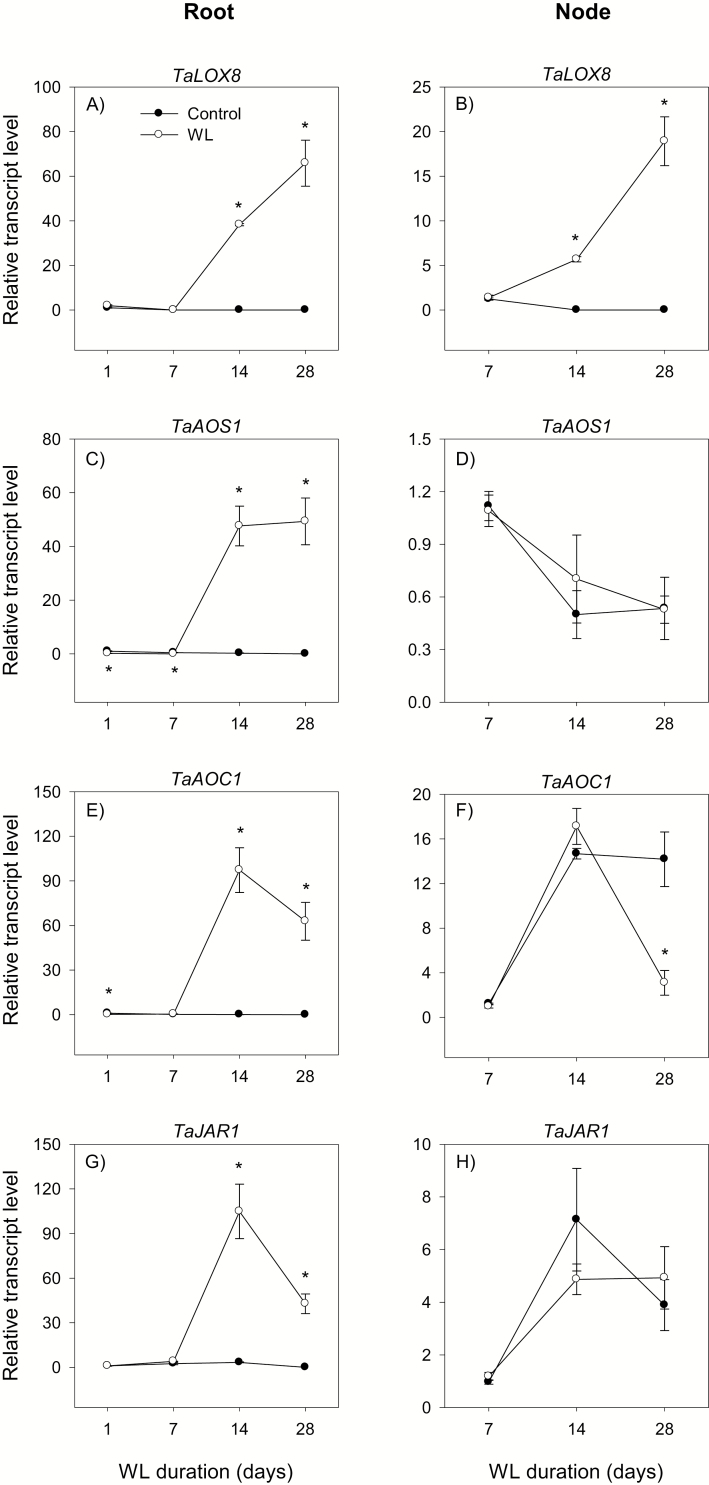
Expression of jasmonate metabolism genes. Relative transcript levels of *LOX8* (A and B), *AOS1* (C and D), *AOC1* (E and F), and *JAR1* (G and H) in the root tissue waterlogged for 1, 7, 14, and 28 d (A, C, E, and G) and stem node tissue waterlogged for 7, 14, and 28 d (B, D, F, and H), and their respective controls. Transcript levels of each gene in both tissues were determined as described in Fig. 3, and are expressed relative to their respective transcript levels in control roots at 1 d after the start of waterlogging, which were arbitrarily set a value of 1. Other data descriptions are as indicated in Fig. 3. *LOX2* in both tissues, *AOS2* in the root, and *AOC2* in the stem node exhibited either relatively lower levels of expression than their respective homologs or undetectable levels of expression irrespective of growth conditions, as shown in Supplementary Table S3.

The expression levels of all the JA metabolic genes expressed in the root were either constantly very weak or decreased below detectable levels by the end of the experiment under control conditions but increased (>3-fold) by waterlogging for 14 d and 28 d (Fig. 8A, C, E, G; Supplementary Table S3). Despite its transient increase and decrease at 7 DAWL in control and waterlogged roots, respectively, the JA-Ile level decreased under control conditions but increased by waterlogging for 14 d and 28 d, leading to the detection of a higher amount of JA-Ile (1.5- to 3.3-fold) in waterlogged than control roots (Fig. 6G).

#### Stem node

The expression of *LOX8* and *AOS1* decreased to low or below detectable levels during the course of the experiment under control conditions, whereas the expression levels of *AOC1* and *JAR1* increased (Fig. 8B, D, F, H). However, the expression level of *LOX8* increased markedly (from an undetectable level) by waterlogging for 14 d and 28 d, while that of *AOC1* decreased in response to waterlogging for 28 d (>4-fold). Waterlogging did not affect the expression levels of *AOS1* and *JAR1*. The stem node JA-Ile level increased under both control and waterlogged conditions, and it was not affected by waterlogging (Fig. 6H).

### Regulation of abscisic acid metabolism

ABA is synthesized from zeaxanthin involving several reactions, and the reaction catalyzed by 9-*cis*-epoxycarotenoid dioxygenase (NCED) is considered to be rate limiting, while the ABA 8′-hydroxylation reaction, which is catalyzed by ABA 8'-hydroxylase (ABA8′OH), represents the major mechanism of ABA inactivation (Nambara and Marion-Poll, 2005). Thus, genes encoding NCED and ABA8′OH (encoded by *CYP707A* genes) play key roles in regulating ABA levels in plant tissues. This study examined the expression patterns of *NCED* (*NCED1* and *NCED2*) and *CYP707A* (*CYP707A1* and *CYP707A2*) genes in root and stem node tissues under control and waterlogged conditions.

#### Root

**Fig. 9.**
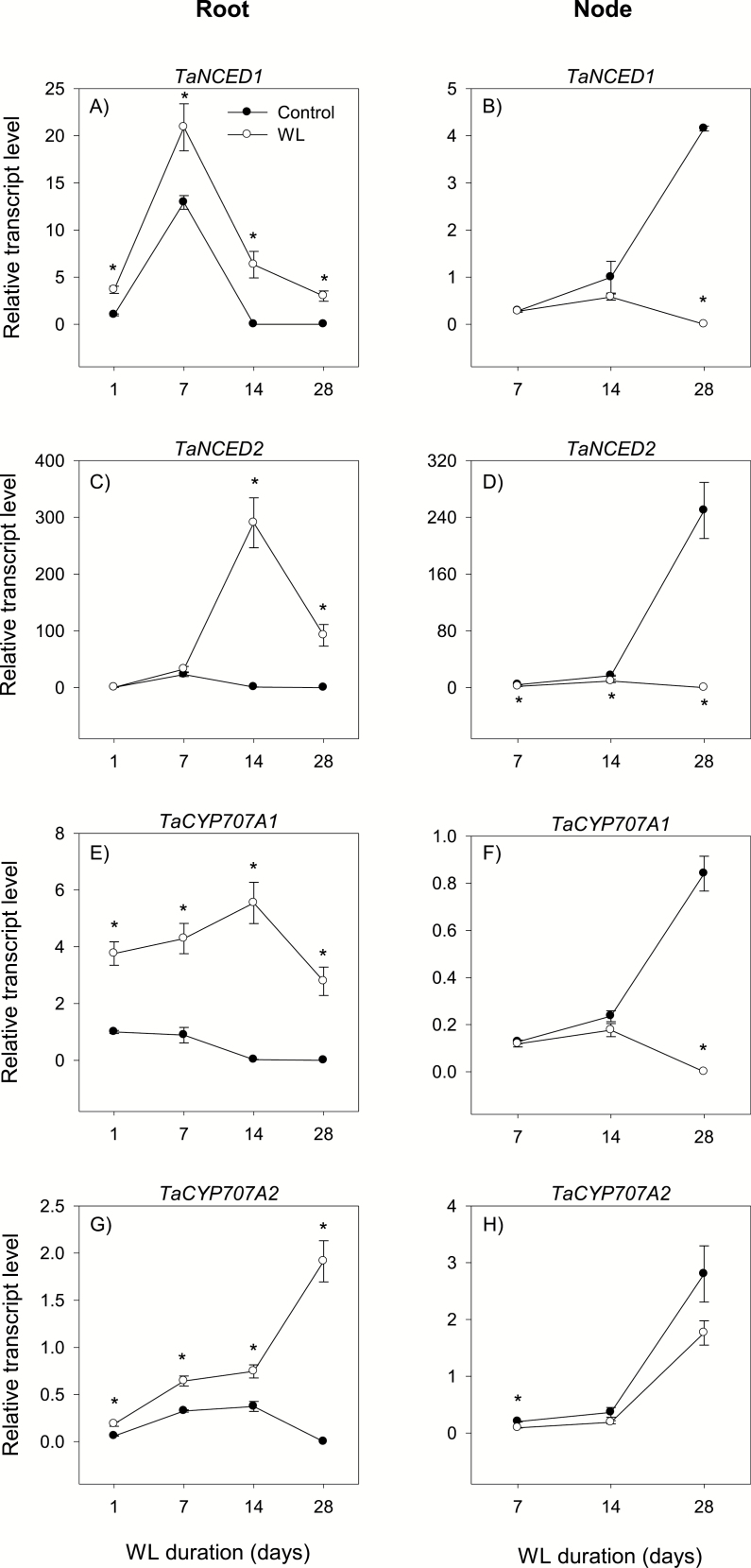
Expression of abscisic acid metabolism genes. Relative transcript levels of *NCED1* (A and B), *NCED2* (C and D), *CYP707A1* (E and F), and *CYP707A2* (G and H) in the root tissue waterlogged for 1, 7, 14, and 28 d (A, C, E, and G) and stem node tissue waterlogged for 7, 14, and 28 d (B, D, F, and H), and their respective controls. Transcript levels of the *NCED* and *CYP707A* genes in both tissues were determined as described in Fig. 3, and are expressed relative to the transcript levels of *NCED1* and *CYP707A1* in control roots at 1 d after the start of waterlogging, respectively, which were arbitrarily set a value of 1. Other data descriptions are as indicated in Fig. 3.

**Fig. 10.**
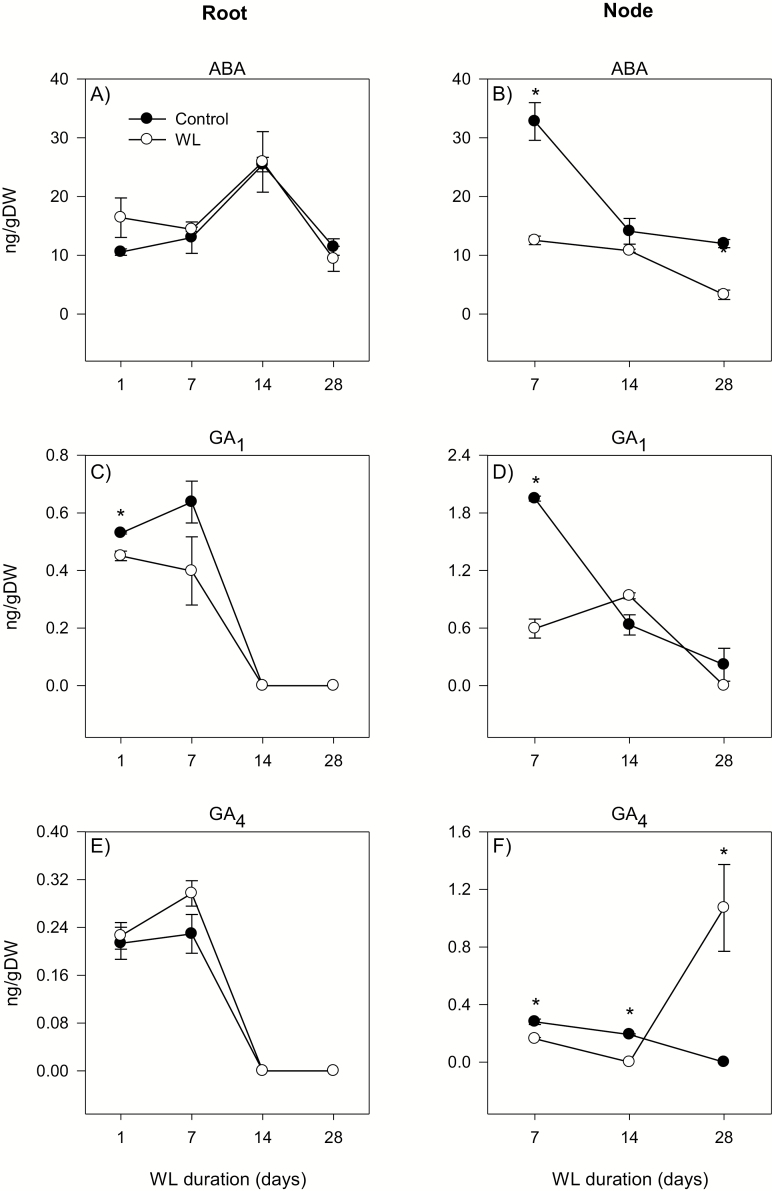
Abscisic acid and gibberellin levels. ABA (A and B), GA_1_ (C and D), and GA_4_ (E and F) levels in the root tissue waterlogged for 1, 7, 14, and 28 d (A, C, and E) and stem node tissue waterlogged for 7, 14, and 28 d (B, D, and F), and their respective controls. Other data descriptions are as indicated in Fig. 6.

The expression of *NCED* and *CYP707A* genes in the root decreased below detectable levels by the end of the experiment under control conditions, except for the transient increases of *NCED1* and *NCED2* expression at 7 DAWL (Fig. 9A, C, E, G). Waterlogging increased the expression level of *NCED1* (from an undetectable level or >1.6-fold) irrespective of the duration and that of *NCED2* (from very low/undetectable level) only after 14 d and 28 d (Fig. 9A, C). Between the two *NCED* genes, *NCED2* exhibited the highest level of waterlogging-induced expression. Waterlogging also increased the expression levels of *CYP707A1* and *CYP707A2* (from undetectable levels or >2-fold) irrespective of the duration (Fig. 9E, G). The root ABA level remained almost constant under both control and waterlogged conditions, except for the transient increase observed at 14 DAWL, and it was not affected by waterlogging (Fig. 10A).

#### Stem node

The expression levels of *NCED* and *CYP707A* genes in the stem node increased during the course of the experiment under control conditions (Fig. 9B, D, F, H). Waterlogging for 28 d, however, decreased the expression of all genes below detectable levels except that the *CYP707A2* expression level showed only a slight reduction (1.6-fold). The stem node ABA level decreased under both control and waterlogged conditions; however, waterlogged stem nodes contained only 27–76% of the ABA found in the corresponding controls (Fig. 10B).

### Regulation of gibberellin metabolism

Gibberellins are synthesized from geranyl geranyl diphosphate, which is converted to *ent*-kaurene by the consecutive actions of *ent*-copalyl diphosphate synthase and *ent*-kaurene synthase (Yamaguchi, 2008). Subsequent oxidation of *ent*-kaurene by *ent*-kaurene oxidase (KO) and *ent*-kaurenoic acid oxidase (KAO) produces GA_12_. The synthesis of bioactive GAs, mainly GA_4_ and GA_1_, involves a series of oxidations of GA_12_ or its 13-hydroxylated form (GA_53_) by the actions of GA 20-oxidase (GA20ox) and GA 3-oxidase (GA3ox), and the bioactive GAs are inactivated mainly through GA 2-oxidation, which is catalyzed by GA 2-oxidase (GA2ox) (Hedden and Phillips, 2000). These enzymes play key roles in regulating bioactive GA levels in plant tissues (Yamaguchi, 2008). This study investigated the expression patterns of GA biosynthesis (*KO*, *GA3ox2*, and *GA3ox3*) and catabolism (*GA2ox8*) genes in waterlogged root and stem node tissues and their respective controls. Our analysis showed that all the GA metabolic genes studied except *GA3ox3* are expressed in both tissues under control and/or waterlogged conditions (Supplementary Table S3).

#### Root

**Fig. 11.**
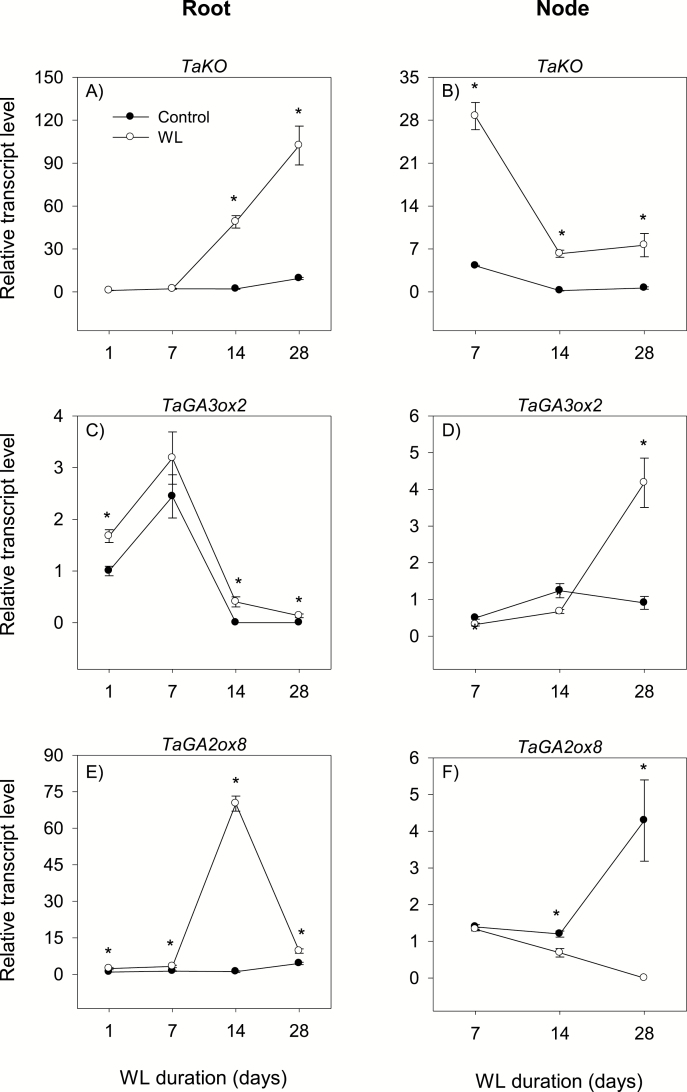
Expression of gibberellin metabolism genes. Relative transcript levels of *KO* (A and B), *GA3ox2* (C and D), and *GA2ox8* (E and F) in the root tissue waterlogged for 1, 7, 14, and 28 d (A, C, and E) and stem node tissue waterlogged for 7, 14, and 28 d (B, D, and F), and their respective controls. Transcript levels of each gene in both tissues were determined as described in Fig. 3, and are expressed relative to their respective transcript levels in control roots at 1 d after the start of waterlogging, which were arbitrarily set a value of 1. Other data descriptions are as indicated in Fig. 3. No transcript of *TaGA3ox3* was detected irrespective of tissue type or growth conditions, as shown in Supplementary Table S3.

The expression levels of *KO*, *GA3ox2*, and *GA2ox8* in the root either remained constantly very weak or decreased below detectable levels during the course of the experiment under control conditions, except for the transient increases of *KO* and *GA2ox8* expression at 28 DAWL (Fig. 11A, C, E). Waterlogging for 14 d and 28 d increased the expression levels of *KO* (10- to 24-fold), *GA3ox2* (from an undetectable level), and *GA2ox8* (2.1- to 62-fold). The waterlogging-induced expression level of *GA2ox8* was, however, much higher than that of *GA3ox2* (Supplementary Table S4). Root GA_1_ and GA_4_ decreased below detectable levels by 14 DAWL under both control and waterlogged conditions, and were not affected by waterlogging (Fig. 10C, E).

#### Stem node

The expression levels of *KO*, *GA3ox2*, and *GA2ox8* in the stem node either decreased or remained constantly weak during the course of the experiment under control conditions, except for the transient increase of *GA2ox8* expression at 28 DAWL (Fig. 11B, D, F). Relative to that observed in the control stem node, waterlogging increased the expression level of *KO* irrespective of the duration (6.7- to 31-fold), while the expression level of *GA3ox2* increased only at 28 DAWL (4.6-fold). In contrast, waterlogging for 14 d and 28 d decreased the expression level of *GA2ox8* (1.8-fold or to an undetectable level). The levels of GA_1_ and GA_4_ in the stem node decreased under both control and waterlogged conditions, except for the transient increase of the GA_4_ level (from an undetectable level to 1 ng g DW^–1^) in response to waterlogging for 28 d (Fig. 10D, F).

## Discussion

Waterlogging decreased the dry weight of the whole root, and this occurred earlier than its effect on the other aspects of root morphology (Fig. 1). It also inhibited elongation of axile roots and emergence of branch/lateral roots while promoting the formation of surface adventitious roots and aerenchyma (Figs 1, 2). Similar effects of waterlogging have also been reported in wheat seedlings and adult plants (Malik *et al.*, 2002; Yamauchi *et al.*, 2014; Herzog *et al.*, 2016). Waterlogging increased the number of axile roots per plant; however, this result contrasts with a previous report that has shown waterlogging-induced reduction in the number of these roots (Malik *et al.*, 2002). This difference could be due to the fact that the two studies used different genotypes or it could arise as a consequence of the growth stages at which the waterlogging treatment was initiated, when the plants were 21 d old (Malik *et al.*, 2002) vs. 30 d old (this study), which potentially affects the establishment and final number of the tillers. Consistently, tiller number was reduced only by 1.5- to 2.3-fold in our study (Fig. 1G) but by up to 4-fold in Malik *et al.* (2002).

The effects of waterlogging on root growth, emergence and elongation of axile and lateral roots, and aerenchyma formation are associated with enhanced expression levels of ethylene biosynthetic genes, mainly *ACS7* and *ACO2* (Fig. 3), suggesting increased ethylene synthesis in waterlogged roots. Consistently, increases in the *ACS* expression level and ethylene synthesis have been reported to be associated with inhibition of root elongation, and the emergence and elongation of branch/lateral roots and promotion of aerenchyma formation in wheat seedlings under oxygen-deficient conditions (Yamauchi *et al.*, 2014). Our results therefore imply the importance of transcriptional control of ethylene synthesis in the root, mainly through the expression of *ACS7* and *ACO2*, in modulating the adaptive response of wheat to waterlogging. Since ethylene also promotes adventitious root emergence in wheat under oxygen-deficient conditions (Yamauchi *et al.*, 2014), our gene expression data in the stem node suggest the significance of transcriptional regulation of ethylene synthesis, mainly via the expression of *ACS7* and *ACO2*, in the formation of surface adventitious roots (Figs 1E, 3).

Previous studies in rice have shown the significance of RBOH-mediated ROS production in ethylene-induced death of epidermal cells during adventitious root emergence from stem nodes (Steffens *et al.*, 2012; Gonzali *et al.*, 2015; Mittler, 2017) and ethylene-induced death of cortical cells during aerenchyma formation under oxygen-deficient conditions (Yamauchi *et al.*, 2017). Consistently, co-induction of the expression levels of ethylene biosynthesis and *RBOH* genes in response to waterlogging is associated with the formation of surface adventitious roots and aerenchyma (Figs 1E, 2–4). It is important to note here that ethylene signaling can also induce epidermal cell death and thereby adventitious root emergence through a ROS-independent signaling pathway (Steffens and Sauter, 2009). Furthermore, the mechanisms underlying the regulation of ethylene synthesis in wheat, one of the dryland crop species, can be different from that of rice (Yamauchi *et al.*, 2015, 2016). Since the role of ethylene in mediating adaptations to flooding/waterlogging/oxygen-deficient conditions has been studied extensively in different crop species including wheat (Jackson *et al.*, 1985; Jackson and Armstrong, 1999; Yamauchi *et al.*, 2013, 2014), our study was focused mainly on the other hormones that are much less studied with respect to waterlogging.

Auxin has been implicated in the inhibition of primary root elongation and promotion of adventitious root formation in cereal crops such as rice (Zhuang *et al.*, 2006; Liu *et al.*, 2009; McSteen, 2010). Therefore, the association of a higher root IAA level (4.1- to 5.1-fold) and shorter axile root phenotype in waterlogged plants with enhanced expression levels of *TDC*, *YUC1*, and *PIN9* (Figs 1, 5, 6) suggests the importance of IAA biosynthesis and transport in regulating the IAA level and root elongation under waterlogged conditions. An increase in IAA level has also been reported in the roots of *Citrus* spp. under long-term flooding (Arbona and Gómez-Cadenas, 2008). The prevalence of a higher IAA level (2.6- to 29-fold) and enhanced expression levels of *TDC*, *YUC1*, and *PIN9* in waterlogged stem nodes is associated with the formation of surface adventitious roots from the stem nodes at 28 DAWL. This, along with the observation of more belowground axile roots in waterlogged than control plants, supports that increased synthesis/transport of auxin positively regulates adventitious root emergence (Boerjan *et al.*, 1995; Liu *et al.*, 2009; Vidoz *et al.*, 2010). Consistently, chemical treatments or mutations that alter auxin transport and synthesis affect adventitious root formation (Liu *et al.*, 2009; Vidoz *et al.*, 2010; Della Rovere *et al.*, 2013; Sauter and Steffens, 2014). Although the *PIN* genes of Arabidopsis act jointly in regulating auxin distribution and thereby determination of root stem cell specification/patterning and growth of primary roots (Blilou *et al.*, 2005), our expression data suggest that *PIN9* acts as a major player in this regard. Increases in the expression levels of IAA-conjugating genes, *GH3.1* and *GH3.2*, in waterlogged roots and stem nodes (Fig. 5) might suggest their negative feedback regulation due to waterlogging-induced IAA accumulation in order to maintain IAA homeostasis. Auxin synthesis in the root and its transport towards the elongation zone where it acts to inhibit cell elongation are stimulated by ethylene (Růžička *et al.*, 2007). Furthermore, auxin transport and adventitious root formation under waterlogged conditions are enhanced by increased ethylene synthesis (Vidoz *et al.*, 2010). Therefore, co-inductions in the expression levels of specific auxin biosynthesis and/or transport and ethylene biosynthesis genes in waterlogged roots and stem nodes (Figs 3, 5) might suggest the importance of auxin–ethylene interplay in regulating root elongation and the formation of surface adventitious roots from the stem nodes.

Cytokinin has been reported to inhibit root elongation in wheat under normal conditions (Stenlid, 1982). Waterlogging increased the expression levels of genes involved in cytokinin synthesis, activation, and inactivation, leading to no significant change in root IPA and *t*-zeatin levels (Figs 6, 7). Thus, inhibition of root elongation under waterlogging conditions with no apparent change in root IPA and *t*-zeatin levels might suggest that the regulation of this root trait in waterlogged wheat is not dependent on the level of cytokinin. Cytokinins are mainly produced in the root and are transported to the shoot (Müller and Leyser, 2011). Consistently, increasing cytokinin synthesis specifically in the root has been shown to improve shoot growth under stress conditions (Ghanem *et al.*, 2011). Given that waterlogging did not affect root IPA and *t*-zeatin levels, the reduced shoot growth in waterlogged plants (Fig. 1F) might be due to impaired root to shoot transport of cytokinin. In agreement with this, short-term flooding has been shown to decrease the level of cytokinin in the xylem sap of sunflower plants (Burrows and Carr, 1969). Cytokinin has also been implicated as a negative regulator of adventitious root formation (Ramírez-Carvajal *et al.*, 2009). Thus, the association of waterlogging-induced reductions in stem node IPA and *t*-zeatin levels with decreased expression levels of *IPT5-2* and *LOG1*, and enhanced expression levels of *CKX5* and *ZOG2* at the earlier stage of waterlogging (by 7 DAWL) (Figs 1E, 2, 6, 7) might suggest the importance of transcriptional regulation of cytokinin biosynthesis, activation, and inactivation in the formation of surface adventitious roots. Given that inhibition of adventitious root formation by cytokinin is linked to its role in repressing the expression levels of auxin transport genes (Della Rovere *et al.*, 2013), the lower cytokinin level in waterlogged stem nodes (Fig. 6D, F) might contribute to the higher expression level of *PIN9* and thereby promoting the formation of surface adventitious roots (Figs 1E, 5J).

Increasing the JA level through mutations or exogenous JA application has been shown to lead to inhibition of root growth in cereals such as rice (reviewed in Liu *et al.*, 2015). Consistently, waterlogging-induced inhibition of root growth is accompanied by an increase in root JA-Ile level, which is associated with enhanced expression levels of JA biosynthetic genes (Figs 6G, 8). It has been shown previously that JA enhances adventitious root formation in rice under normal/aerobic conditions (Moons *et al.*, 1997). However, the emergence of surface adventitious roots from stem nodes waterlogged for 28 d in spite of the absence of significant effects of waterlogging on the expression level of *JAR1*, a gene encoding an enzyme catalyzing the final step of JA-Ile synthesis, and JA-Ile level in the stem node (Figs 1E, 6H, 8H) suggests that this root morphological acclimation to waterlogging in wheat is not regulated by the JA level.

ABA is also implicated in the inhibition of primary root elongation in cereal crops such as rice under normal conditions (Konishi *et al.*, 2005), although it has the opposite effect under stress conditions such as drought/low water potential (Saab *et al.*, 1990; Sharp *et al.*, 1994). Waterlogging enhanced the expression levels of both ABA biosynthetic (*NCED*) and catabolic (*CYP707A*) genes in the root relative to that observed under control conditions, leading to no change in ABA level. The prevalence of shorter roots in waterlogged wheat with no apparent effect on root ABA level (Figs 1, 10A) suggests that inhibition of wheat root elongation under waterlogging conditions is not dependent on the ABA level. Previous studies with other plant species such as alfalfa and Arabidopsis have also shown that the root ABA level is not affected by long-term flooding/oxygen-deficient conditions (Castonguay *et al.*, 1993; de Bruxelles *et al.*, 1996). The emergence of surface adventitious roots from waterlogged stem nodes along with reductions in the expression levels of ABA biosynthetic genes and stem node ABA content (Figs 1E, 9B, D, 10B) support that ABA acts as a negative regulator of adventitious root formation (Steffens and Sauter, 2005).

Although root bioactive GA decreased below detectable levels in the course of the experiment under both control and waterlogged conditions (Fig. 10C, E), the higher expression level of the GA catabolic gene, *GA2ox8*, relative to that observed for the regulatory GA biosynthetic gene, *GA3ox2*, in roots waterlogged for 14 d and 28 d (Supplementary Table S4) might suggest lower root GA levels that cause inhibition of root elongation as observed in rice (Komatsu and Konishi, 2005). Based on the detection of a reduced GA level in the xylem sap, Reid *et al.* (1969) also suggested decreased GA synthesis in waterlogged roots of tomato. Previous reports have implicated GA in promoting ethylene-induced adventitious root formation (Suge, 1985; Steffens and Sauter, 2005). Thus, the association of waterlogging-induced accumulation of bioactive GA (GA_4_) with the expression patterns of GA biosynthesis (*KO* and *GA3ox2*) and inactivation (*GA2ox8*) genes in stem nodes that produce surface adventitious roots (Figs 1E, 10F, 11) implies the importance of transcriptional control of GA metabolism in regulating this morphological acclimation of wheat to waterlogging.

In summary, the results of our study suggest that morphological and anatomical adaptive responses of wheat to waterlogging involve modulation of the levels of different plant hormones in root and stem node tissues through transcriptional regulation of specific genes in their respective metabolic pathways, and the findings of this study provide important insights into the molecular mechanisms underlying the acclimation of wheat to waterlogging.

## Supplementary data

Supplementary data are available at *JXB* online.

Table S1. Sequence similarity of genes analyzed in this study with their orthologs.

Table S2. Gene-specific primers used for expression analysis.

Table S3. Expression levels of hormone metabolic genes analyzed in this study.

Table S4. Comparison of expression levels across all gibberellin metabolism genes.

Supplementary Table S1

Supplementary Table S2

Supplementary Table S3

Supplementary Table S4

## Abbreviations:

ABA: abscisic acid
DAWL: days after waterlogging
GA: gibberellin
IAA: indole acetic acid
JA: jasmonate
JA-Ile: jasmonoyl-l-isoleucine
ROS: reactive oxygen species.
